# The impact of intracanal medicaments on crown color, pH, and antimicrobial activity: a comparative study

**DOI:** 10.1590/1807-3107bor-2025.vol39.029

**Published:** 2025-02-27

**Authors:** Patrícia Correia de SIQUEIRA, Lorrayne da Costa Cassimiro SOARES, Keryda Ramos Di Machado WANDERLEY, Lara Borges de DEUS, Higor Venâncio de MELO, Maria Paula Andrade ÁVILA, Lucas Rodrigues de Araújo ESTRELA, Cyntia Rodrigues de Araújo ESTRELA

**Affiliations:** (a) Universidade Federal de Goiás – UFG, School of Dentistry, Department, Goiânia, GO, Brazil.; (b) Universidade Evangélica de Goiás, School of Dentistry, Department, Anápolis, GO, Brazil.

**Keywords:** Calcium Hydroxide, Calcarea Silicata, Dental Materials, Root Canal Therapy

## Abstract

This study aimed to compare the effects of intracanal medication pastes Bio-C Temp® (BC), UltraCal XS® (UC), and Pure Calcium Hydroxide (CH) on crown color change, pH, and antimicrobial activity. Color change was assessed using the CIEL*a*b* color space system parameter. pH values were measured at 3, 24, 72, and 168 hours. Antimicrobial activity was evaluated using agar diffusion and direct exposure tests. Statistical analyses were conducted using ANOVA, Kruskal-Wallis, and Friedman tests (α = 0.05). After 15 days, BC exhibited significantly greater color variation than the control, and higher L* values at 60 days compared to baseline (p < 0.05). BC demonstrated lower pH values than UC and CH at 3 and 72 hours (p < 0.05). In the agar diffusion test, BC produced a smaller microbial inhibition halo than UC and CH. In the direct exposure test, CH completely inhibited microbial growth, whereas BC showed microbial growth at all evaluated time points. In conclusion, BC resulted in greater crown color change, lower pH, and reduced antimicrobial activity compared to UC and CH.

## Introduction

The disinfection process achieved during root canal preparation significantly reduces microorganisms within the canal, but does not entirely eliminate them. The survival of microorganisms in the root canal system can contribute to endodontic treatment failure and the development or persistence of apical periodontitis, since these bacteria are shielded from host immune responses and systemic antibiotics.^1^ Consequently, effective treatment strategies should focus on maximizing microbial reduction through the combined mechanical and physicochemical action of endodontic instruments, irrigating solutions, and intracanal medication, followed by appropriate endodontic sealing. Intracanal medications play a crucial role in targeting residual microorganisms within areas of anatomical complexity and dentinal tubules, which are difficult to access during mechanical preparation. By achieving deeper penetration and enhanced disinfection, these medications can improve the success rates of endodontic treatment.^2^

Calcium hydroxide has been the most widely used and recommended intracanal medication for decades due to its antimicrobial potential and its biological effect of inducing mineralization.^3^ Its mechanism of action involves ionic dissociation into calcium and hydroxyl ions upon contact with body tissues.^4^ The high pH resulting from hydroxyl ion release disrupts the structural integrity of bacterial cytoplasmic membranes and activates alkaline phosphatase. Furthermore, calcium ions react with tissue carbon dioxide, promoting mineralization. Calcium hydroxide also degrades bacterial lipopolysaccharides, contributing to its antimicrobial efficacy.^1,4^Various ready-to-use CH paste formulations, such as UltraCal XS®, facilitate application into the root canal. These formulations often include radiopacifying agents, enabling visualization during imaging exams.^5,6^

Recently, a calcium silicate-based intracanal medication, Bio-C Temp®, was developed. Calcium silicate-based materials, commonly referred to as bioceramics or hydraulic cements, are derived from mineral trioxide aggregate (MTA), and have been widely utilized in endodontics for various clinical applications. Based on their consistency, these materials can also be employed as repair agents or as endodontic sealers for root canal obturation. While studies have demonstrated their satisfactory biocompatibility and effective sealing properties, concern persists regarding their potential to cause tooth discoloration following clinical application.^7^ Since one of the essential properties of intracanal medication is its ability to avoid tooth discoloration, aesthetic considerations play a crucial role in selecting a material for this purpose.

Although studies have assessed the properties of this new calcium silicate-based medication,^8-14^ few have directly compared it with Pure Calcium Hydroxide. Consequently, further investigations are required to validate the desired properties of intracanal medications. This study aimed to evaluate and compare the crown color change, pH, and antimicrobial activity of three intracanal medication pastes: Bio-C Temp®, UltraCal XS®, and Pure Calcium Hydroxide. The null hypothesis tested was that no differences exist in these properties among the materials evaluated.

## Methods

The materials evaluated in this study were Bio-C Temp® (Angelus, Londrina, Brazil), UltraCal XS® (Ultradent, Indaiatuba, Brazil), and Pure Calcium Hydroxide (Biodinâmica, Ibiporã, Brazil). The chemical compositions provided by the manufacturers are detailed in Table 1.

**Table 1:** Commercial names, chemical compositions, manufacturers, and batch numbers of the medications evaluated in this study.

Material	Chemical composition	Manufacturer / Batch Number
Bio-C Temp®	Tricalcium Silicate, Dicalcium Silicate, Tricalcium Aluminate, Calcium Oxide, Base Resin, Calcium Tungstate, Polyethylene Glycol, Titanium Oxide	Angelus, Londrina, PR, Brazil / Batch: 60011

### Evaluation of Crown Color Change

Evaluations of color change were based on a sample size calculation derived from data in a previous study.^9^ The calculation determined that 12 teeth per group were required, with a significance level of 5%, 80% statistical power, a minimum detectable difference of 3.3, and an estimated standard deviation of 2.4. A total of 48 intact bovine central incisors were collected from a certified slaughterhouse following good animal practices (Zanchetta Indústria de Alimentos, Bauru, Brazil). The teeth were immersed in 2.5% sodium hypochlorite (Fitofarma, Goiânia, Brazil) for 1 hour to remove organic tissues, and subsequently stored in a flask containing 0.2% thymol solution (University Pharmacy-UFG, Goiânia, Brazil). Digital periapical radiographs were taken to assess eligibility, excluding teeth with cracks, fractures, calcifications, internal resorptions, and root canal obliterations.

The roots of the teeth were measured with a digital caliper and sectioned 5 mm below the cementoenamel junction using a diamond disc at low speed under refrigeration (Komet, Besigheim, Germany). The crowns were then randomly divided into four groups (n = 12): three experimental groups, each corresponding to one material, and a control group with no material inserted into the root canal. Following group allocation, the samples were immersed in distilled water and stored in an incubator at 37°C for 24 hours. Evaluations of baseline color (T0) were conducted after this incubation period using a spectrophotometer (Easyshade®, Vita, Bad Säckingen, Germany). A silicone guide (Reflex, Yller Biomateriais, Pelotas, Brazil) was created for each sample, ensuring consistent positioning of the device for all measurements. The guide featured a hole positioned 2 mm above the cementoenamel junction on the buccal surface of the crown, precisely aligning with the spectrophotometer tip. Measurements were performed in a controlled environment with standardized lighting conditions and a white background, and the spectrophotometer was calibrated before assessing the color of every three specimens.

Sample preparation involved prophylaxis using pumice paste and water with a rubber cup at low speed for 20 seconds on each tooth face. Access to the crowns was achieved with spherical diamond burs (1013 HL, KG Sorensen, Cotia, Brazil) at high speed under refrigeration. Canal preparation was performed using Largo 1 and 2 drills (Microdont, São Paulo, Brazil) with constant irrigation using 1% sodium hypochlorite. Following preparation, the root canals were irrigated with 10 ml of 17% EDTA solution (Biodinâmica Química e Farmacêutica, Ibiporã, Brazil), followed by a final irrigation with 10 ml of distilled water. The root canals were dried with paper points, and the apices were sealed with type 7 wax (Lysanda, Vila Prudente, Brazil). Bio-C Temp® (BC) and UltraCal XS® (UC) pastes were inserted directly into the root canal according to the manufacturers’ instructions. Pure Calcium Hydroxide (CH) was mixed with saline solution and inserted into the root canal using a #50 K-file (Dentsply Sirona, Ballaigues, Switzerland) and paper points. In the control group, no medication was applied to the root canal. The coronal chamber was cleaned with a saline-soaked cotton ball, and coronal sealing was completed using Herculite Classic composite resin (Kerr, Orange, USA) with the Single Bond Universal adhesive system (3M, St. Paul, USA). At this stage, the color of the dental specimens in all the groups was reassessed (T1) using the same parameters as the initial reading.

The samples were stored in an incubator at 37°C and 100% humidity throughout the experiment. Color measurements were repeated at 15 days (T2), 30 days (T3), and 60 days (T4). Color was assessed using the CIE L*a*b* system parameters, where L* represents luminosity, ranging from 0 (black) to 100 (white), and a* and b* represent hue, with a* indicating saturation on the red-green axis and b*, on the blue-yellow axis. The color coordinates for each sample were measured three times, and the average values were calculated to ensure greater measurement accuracy. Color variation (ΔE*) was determined using the formula: $\Delta E^{*}=\left[{\left(\Delta L^{*}\right)}^{2}+{\left(\Delta a^{*}\right)}^{2}+{\left(\Delta b^{*}\right)}^{2}\right]0.5$ , where: $\Delta L^{*}=L1^{*}-L0^{*}$ ; $\Delta a^{*}=a1^{*}-a0^{*}$ and $\Delta b^{*}=b1^{*}-b0^{*}.\Delta E^{*}$ values ≥ 3.7 were considered clinically perceptible^15^.

### pH Evaluation

The pH evaluation was conducted using polyethylene tubes (10 mm long x 1 mm inner diameter) sealed at one end. The pastes were inserted into the tubes, with five specimens prepared for each experimental time point and each material. Each tube was placed in a 40-mm-diameter container holding 10 ml of distilled and deionized water with a previously measured pH. The samples were stored in an incubator at 37°C. pH measurements were taken after 3, 24, 72, and 168 hours of immersion. Before each measurement, the paste samples were removed from the tubes, and the solutions were manually agitated for 5 seconds. A pH meter (NT-PHM, MS Tecnopon, São Paulo, SP, Brazil) calibrated with buffer solutions at pH 4.0, 7.0, and 10.0 was used for the readings. A control for this method was established by measuring the pH values of deionized water without any sample immersed.

### Antimicrobial Activity

The antimicrobial activity was assessed using an *Enterococcus faecalis* strain (ATCC 29212) as a biological indicator. The strain was inoculated in 7 ml of Brain Heart Infusion (BHI; Difco Laboratories, Detroit, USA) and incubated at 37°C for 24 hours. After incubation, microbial cells were suspended in saline solution to achieve a final concentration of approximately 3 x 10^8^ cells/ml, adjusted to the McFarland 1 turbidity standard.

For the agar diffusion test, 10 Petri dishes containing 20 ml of BHI agar (BHIa; Difco Laboratories, Detroit, USA) were inoculated with 1 ml of microbial suspensions using sterilized swabs. The inoculum was spread over the surface of the culture medium to achieve confluent growth. The pastes were deposited into 10 wells created in the contaminated agar plates. No substance was applied to the plate that served as a negative control. The plates were incubated at 37°C for 48 hours. Following incubation, the diameters of the microbial inhibition halos around the materials were measured using a digital caliper. Positive and negative controls were included by maintaining inoculated and non-inoculated BHIa plates, respectively, under identical incubation conditions.

For the direct exposure test, absorbent paper points (#50; Tanari, Tanariman, Manacapuru, Brazil) were sterilized and immersed in the microbial suspension for 5 minutes (n = 3 per material). The paper points were then placed in Petri dishes and covered with the respective pastes for incubation periods of 7, 14, and 21 days at 37ºC. At the end of each incubation period, the points were removed from the pastes and individually transferred to 5 ml of Letheen Broth (Difco Laboratories, Detroit, USA). After 48 hours of incubation at 37°C, 1 ml of the broth was transferred to 5 ml of BHI and incubated for another 48 hours under the same conditions. Microbial growth was assessed by the turbidity of the medium, determined through spectrophotometric readings. All experiments were performed under aseptic conditions.

### Statistical Analysis

The results were tabulated and analyzed using Jamovi software (version 1.2.27), with a significance level set at 5%. Data distribution was assessed using the Shapiro-Wilk test. The results from the crown color change, pH, and agar diffusion tests followed a parametric distribution and were analyzed using one-way ANOVA followed by Tukey’s test for multiple comparisons.

Data from the direct exposure test exhibited a non-parametric distribution and were analyzed using the Kruskal-Wallis test for independent samples and the Friedman test for dependent samples.

## Results

### Evaluation of Crown Color Change

The ∆E* results for color change at the evaluated time points are shown in Table 2. After 15 days, a significant difference was observed between the BC group and the control group (p < 0.05), with the BC group exhibiting greater color variation. However, no significant differences were observed among the groups after 30 and 60 days (p>0.05). Similarly, no significant differences in ∆E* values were found when comparing different time intervals within each group (p > 0.05). Clinically perceptible color changes (∆E* ≥ 3.7) were observed at some time points for all groups except for the control group.

**Table 2:** Mean and standard deviation (SD) values of ∆E* (color change) for each group at the evaluated time points.

Group	∆E* 15 days	∆E* 30 days	∆E* 60 days
BC	4.53 ±2.98 ^A,a^	4.83 ±2.74 ^A,a^	4.53 ±2.90 ^A,a^
UC	3.38 ±1.27 ^AB,a^	3.05 ±1.61 ^A,a^	4.38 ±2.88 ^A,a^
CH	3.01 ±2.16 ^AB,a^	2.92 ±1.48 ^A,a^	3.75 ±1.94 ^A,a^
Control	2.06 ±1.23 ^B,a^	2.78 ±1.53 ^A,a^	2.64 ±1.44 ^A,a^

Different uppercase superscript letters indicate significant differences between groups for each time interval (columns), and different lowercase superscript letters indicate significant differences between time points within each group (rows), determined by ANOVA and Tukey’s multiple comparisons test.

The L* values at different experimental times are presented in Figure. No statistically significant differences were observed among the groups at each experimental time based on the Kruskal-Wallis test (p > 0.05). However, the BC group exhibited significantly higher L* values at 15, 30, and 60 days compared to the baseline reading, as determined by the Friedman test (p < 0.05).

**Figure:**
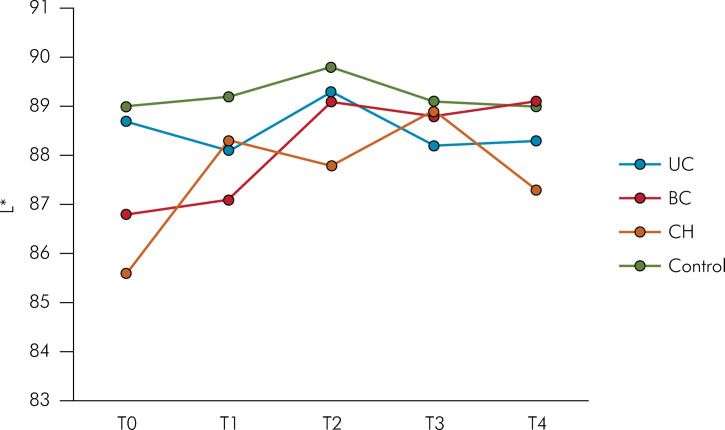
Median L* values per group across experimental time points: T0 (baseline); T1 (initial); T2 (15 days); T3 (30 days); T4 (60 days). UC: UltraCal XS; BC: Bio-C Temp; CH: Pure Calcium Hydroxide.

### pH Evaluation

The pH values for each group are summarized in Table 3. After 3 and 72 hours, BC exhibited significantly lower pH values compared to UC and CH (p < 0.05). There was no difference between UC and CH during these periods. After 24 hours, no pH differences were detected among the medications (p > 0.05). However, after 168 hours, CH showed a significantly higher pH than both BC and UC (p < 0.05). Analyzing the pH changes within each medication over time, BC and CH demonstrated no significant differences across the four experimental time points (p > 0.05). However, UC showed a significant reduction in pH after 168 hours (p < 0.05).

**Table 3:** Mean and SD values of pH for each experimental group.

Group	3h	24h	72h	168h
BC	7.70 (± 0.27)^a, A^	8.56 (± 0.19)^a, A^	7.70 (± 0.27)ª^, A^	7.78 (± 0.50)ª^, A^
UC	9.69 (± 0.53)^b, AB^	9.32 (± 0.53)^a, AB^	10.3 (± 0.11)^b, A^	8.39 (± 0.44)ª^, B^
CH	9.48 (± 0.35)^b, A^	9.94 (± 0.20)ª^, A^	10.4 (± 0.06)^b, A^	10.3 (± 0.13)^b, A^

Different lowercase superscript letters indicate significant differences between medications for each time point (columns), while different uppercase letters represent significant differences between time points for each medication (rows), determined by ANOVA and Tukey’s multiple comparisons test.

### Antimicrobial Activity

The results of the agar diffusion and direct exposure tests are presented in Tables 4 and 5, respectively. In the agar diffusion test, BC produced significantly smaller inhibition halos compared to UC and CH. In the direct exposure test, CH showed no microbial growth at any evaluated interval, UC demonstrated growth at 14 days, and BC exhibited microbial growth at all time points. Despite these observations, there were no significant differences among the groups in the direct exposure test.

**Table 4:** Mean and SD values of inhibition halo diameters (mm) from the agar diffusion test.

Group	Mean (SD)
BC	10.3 (0.95)^a^
UC	13.7 (0.82)^b^
CH	14.1 (0.88)^b^

Different superscript letters indicate statistically significant differences, determined by ANOVA and Tukey’s multiple comparisons test.

**Table 5:** Median and interquartile range (1st and 3rd quartiles) values of medium turbidity obtained by spectrophotometer for each group at the evaluated time points in the direct contact test.

Group	7 days	14 days	21 days
BC	0.010^a^ (0.005/0.183)	0.011^a^ (0.008/0.318)	0.010^a^ (0.009/0.014)
UC	0.000^a^ (0.000/0.000)	0.018^a^ (0.009/0.031)	0.000^a^ (0.000/0.000)
CH	0.000^a^ (0.000/0.000)	0.000^a^ (0.000/0.000)	0.000^a^ (0.000/0.000)

Different superscript letters indicate statistically significant differences, determined by the Kruskal-Wallis test.

## Discussion

The findings of this study support the rejection of the null hypothesis, since significant differences were identified in color change, pH, and antimicrobial activity among the tested materials. All intracanal medications evaluated caused clinically perceptible color change (ΔE* ≥ 3.7) during at least one of the experimental periods, with the calcium silicate-based paste (BC) exhibiting the greatest variation compared to the control group. Notably, BC demonstrated significant color change at the 15-day evaluation, but no further changes were observed at 30 and 60 days, indicating stability during the latter periods.

Bovine incisors were utilized to evaluate dental color changes, consistent with other studies investigating endodontic materials^9,10,13,16,17^. This experimental model offers several advantages, including ease of obtaining an adequate sample size and the favorable dimensions of bovine teeth. Specifically, the larger and flatter surfaces of bovine teeth, compared to human teeth, facilitate more accurate color evaluations.^16^

The Vita spectrophotometer was employed for color assessment due to its sensitivity to minor color variations and its ability to provide quantitative data. Spectrophotometric measurements of tooth color commonly utilize the CIE L*a*b* system, which was developed by the Commission Internationale de l’Eclairage (CIE), an organization recognized by ISO as an international standardization body for light, vision, and color.^18^ The CIE L*a*b* system is a three-dimensional model that distinguishes colors between objects. In this system, higher L* values indicate greater differences in color, which are consequently more perceptible to the human eye.^13^

ΔE* values provide quantitative data, but do not indicate the direction of color variation along the luminosity axis.^9^ To assess this, the L*, a*, and b* axes are analyzed qualitatively. The threshold for human perceptibility is defined as ΔE* values ≥ 3.7.^15^ Color changes may result from chemical interactions between the components of endodontic materials and dental structures.^17^ The penetration of materials into dentinal tubules and the transmission of color through dentin and enamel remnants have been proposed as potential mechanisms for chromatic alteration.^19^

In contrast to the present findings, Campos et al.^13^ observed chromatic alteration by BC in bovine teeth only after 6 months and 1 year. Despite similarities in sample preparation, methodological differences should be noted. In the referred study, the materials were left in the root canal for 21 days and subsequently removed to evaluate their residual effect on tooth color over longer periods of up to 1 year.

Another study^9^ investigating immediate color changes following material insertion and over 30, 45, and 60 days of follow-up observed that all tested materials caused dental discoloration exceeding the acceptable threshold. BC demonstrated less or comparable color changes relative to MTA Flow® and UC. Furthermore, BC and UC exhibited no significant difference after 60 days, suggesting similar chromatic alterations within the evaluated period. These findings were corroborated by a subsequent study^10^ simulating clinical pulpotomy conditions, where BC and other tested materials were associated with dental discoloration. These results align with those of the present study, confirming the potential for these materials to induce some level of color change.

This study additionally included a comparative analysis of L* values, which represent the degree of luminosity (dark to light), and are considered a primary clinical aesthetic concern. The BC group showed an increase in L* values after 15 days compared to baseline, maintaining stability until 60 days. This suggests that teeth treated with BC became lighter over time. According to the manufacturer, BC contains calcium silicates as the primary components, along with calcium aluminate, calcium oxide, radiopacifiers (calcium tungstate and titanium oxide), and vehicles (base resin and polyethylene glycol). Color changes associated with dental materials are often linked to their chemical composition, particularly the types of radiopacifiers. Pelepenko et al.^20^ similarly reported increased L* values after 28 and 90 days using another calcium silicate-based material, Bio-C Pulpo, in bovine tooth blocks. Unlike BC, Bio-C Pulpo contains zirconium oxide as a radiopacifier.

The incorporation of alternative radiopacifying agents, such as calcium tungstate and zirconium oxide, aims to reduce the risk of dental darkening associated with bismuth oxide in MTA. Studies suggest that these agents do not induce dental darkening.^16,17,21,22^ The migration of calcium tungstate into the dental structure was demonstrated in an in vitro study using element mapping with scanning electron microscopy; however, this migration did not result in tooth color changes.^16^ Nonetheless, some studies have reported clinically perceptible color changes associated with cements containing calcium tungstate, as observed in the present study, which may explain these findings.^13,22^

Several factors contribute to tooth color change following endodontic treatment, including pulp hemorrhage, the materials used, and the technique applied^19^. Modern endodontics must address not only biological and functional aspects, but also aesthetic considerations, since nearly all endodontic materials are associated with some degree of crown color change.^23^ The findings of this study highlight the lack of consensus in the literature, underscoring the need for further investigations to better understand the interactions between radiopacifying agents in calcium silicate-based cements and dentin. Future research should analyze different parameters of color change and evaluate the behavior of these materials under clinical conditions.

All tested materials exhibited alkaline pH; however, BC demonstrated lower pH values compared to UC and CH, consistent with previous studies.^8,12,14^ UC and CH showed similar pH levels, with CH maintaining the highest values even after the longest evaluation period. These findings align with those of Viana et al.,^12^ who reported an average pH of 10.08 for CH after 168 hours. The elevated pH of CH can be attributed to the release of hydroxyl ions upon dissociation, with its higher hydroxyl ion concentration in CH compared to other pastes, likely accounting for the sustained pH values observed even after extended evaluation periods.

An alkaline pH (8.6 to 10.3) is essential for the biological action of intracanal medications, supporting the mineralization process and eliminating microorganisms involved in endodontic infections.^24^ However, a pH above 11 can be cytotoxic to periapical tissues.^14^ Among the materials evaluated, CH was the only one to exhibit satisfactory average pH values throughout all experimental periods. In contrast, BC failed to reach the minimum recommended pH, with its highest value being 8.56 after 24 hours.

BC demonstrated the lowest antimicrobial activity against E. faecalis in both tests performed, corroborating findings by Guerreiro et al.^11^ Another study similarly reported that CH pastes mixed with saline or propylene glycol exhibited superior antibacterial activity compared to UC and BC, which showed no significant differences.^12^ The low antimicrobial activity of BC may be attributed to the reduced formation of calcium hydroxide molecules during the hydration reaction of calcium silicate-based cements.^11^

The vehicles used in intracanal medication pastes influence their chemical dissociation, diffusibility, and filling capacity, which are critical factors in determining their biological behavior.^24^ These pastes also differ in additives and calcium hydroxide proportions, potentially affecting bacterial reduction.^11^ UC is a ready-to-use paste containing approximately 35–36% calcium hydroxide (active ingredient), barium sulfate (radiopacifier), and methylcellulose (vehicle). Its lower calcium hydroxide concentration compared to the pure formulation results in a reduced release of calcium and hydroxyl ions into tissues, consequently diminishing its antibacterial effect. This aligns with the findings of the present study, where UC exhibited less antibacterial activity than CH mixed with saline. BC showed the lowest antibacterial activity among the tested materials, likely due to its lower alkalinity and limited calcium hydroxide formation during hydration.^11^ Given that one of the primary functions of intracanal medication is to enhance root canal system decontamination, complementing the use of instruments and irrigating solutions, its antimicrobial efficacy should be a critical factor in selecting the most appropriate material.

Although laboratory studies provide valuable insights into the properties of materials used in endodontics, the clinical behavior of these materials must be assessed through well-designed clinical studies. Such evaluations are essential to determine their effectiveness within the biological context of their application, taking into account the patient’s systemic and immune responses.

## Conclusions

Bio-C Temp® exhibited greater color change and lower pH values compared to the other pastes. All three tested medications demonstrated antimicrobial activity, with Pure Calcium Hydroxide showing the most favorable results in both tests. Laboratory studies play a critical role in elucidating the specific properties of endodontic materials. These findings provide a foundation for future research and serve as a guide for selecting effective endodontic materials to ensure successful endodontic therapy.
